# Strengthening workforce resilience to sustain community health services during crises: evidence from Chinese general practitioners

**DOI:** 10.3389/fpubh.2025.1633175

**Published:** 2025-07-17

**Authors:** Botang Guo, Yongbin Li, Ying Fu, Minyao Li, Yue Wang, Shuyu Liu, Yanjuan Zhou, Yu Liu, Ping Tang

**Affiliations:** ^1^Department of General Practice, The Affiliated Luohu Hospital of Shenzhen University Medical School, Shenzhen, China; ^2^Department of Allergy and Clinical Immunology, State Key Laboratory of Respiratory Disease, National Clinical Research Center for Respiratory Disease, Guangzhou Institute of Respiratory Health, The First Affiliated Hospital of Guangzhou Medical University, Guangzhou, China; ^3^Department of Physical Examination, Rehabilitation Branch of Luohu District Traditional Chinese Medicine Hospital, Shenzhen, China; ^4^School of Medicine, Harbin Vocational and Technical University, Harbin, China; ^5^Center of Psychological, Harbin First Specialized Hospital, Harbin, China; ^6^College of Marxism, Harbin Medical University, Harbin, China

**Keywords:** organizational resilience, work engagement, turnover intention, community health services, pandemic preparedness

## Abstract

**Background:**

Ensuring the resilience and retention of the primary healthcare workforce is critical to maintaining health service continuity during public health crises. While pandemic preparedness frameworks increasingly emphasize integrated economic and health policies, the role of organizational and psychological factors in sustaining frontline health personnel has received less empirical attention. This study aimed to investigate the prevalence of turnover intention among community general practitioners (GPs) in Luohu District, Shenzhen, China, and to examine the mediating role of work engagement in the relationship between perceived organizational resilience and turnover intention.

**Methods:**

A cross-sectional survey was conducted among 927 community health physicians from Aug to Dec in 2024 in Luohu District, Shenzhen, a region where all community health centers are governed by a unified hospital group. Participants completed validated scales assessing organizational resilience, work engagement, and turnover intention. Descriptive statistics, correlation analysis, and hierarchical regression analysis were performed. The mediating effect of work engagement was tested using bootstrapping procedures, controlling for significant demographic and occupational covariates.

**Results:**

A high prevalence of turnover intention was observed, with 65.16% of GPs reporting high or very high levels. Significant differences in turnover intention were found across age groups, professional titles, cadre status, monthly income, and perceived public health workload. Mediation analysis revealed that work engagement partially mediated the relationship between organizational resilience and turnover intention (*B* = −0.018, 95%CI [−0.070,–0.011] without covariates; adjusted indirect effect *B* = –0.019, 95%CI [−0.026,–0.012] with covariates), accounting for approximately 16.82% (17.12% adjusted) of the total effect. These relationships remained significant after controlling for covariates.

**Conclusion:**

Turnover intention among community GPs in the studied region is alarmingly high, posing a significant challenge to workforce stability. Organizational and psychological resources play a key role in mitigating workforce attrition risks in community health systems. Strengthening organizational resilience and fostering work engagement may offer cost-effective, non-technological strategies to maintain essential health service delivery capacity during future pandemics, aligning with broader goals of economic stability and pandemic preparedness.

## 1 Introduction

### 1.1 The escalating challenge of public health crises and the imperative for workforce resilience

The 21st century has been marked by an increasing frequency and intensity of public health crises, ranging from pandemics like COVID-19 to regional epidemics and a spectrum of natural and man-made disasters (1). These events invariably place immense strain on healthcare systems worldwide, challenging their capacity to deliver essential services and respond effectively to surging demands. Within this context, the resilience of the healthcare system—its ability to prepare for, respond to, and recover from such adversities—has become a paramount concern for global health security (2). A critical, yet often underemphasized, component of this systemic resilience is the resilience and stability of its frontline workforce, particularly those in community health services (3).

Community health services, with general practitioners (GPs) at their core, function as the bedrock of primary healthcare and act as crucial gatekeepers during health emergencies (4). They are instrumental in early detection, triage, managing less severe cases, providing ongoing care for chronic conditions, and alleviating pressure on secondary and tertiary healthcare facilities (5). However, the very nature of their frontline role exposes community healthcare professionals to a maelstrom of stressors during crises (6). These include, but are not limited to, overwhelming workloads, heightened risk of infection, psychological distress stemming from fear and uncertainty, resource scarcity, and sometimes, societal stigmatization (7). Such sustained pressures significantly elevate the risk of burnout, compassion fatigue, and ultimately, the intention to leave their positions or the profession entirely (8). High turnover intention among these vital personnel during a crisis can cripple the continuity of essential health services, exacerbate health inequities, and undermine the overall effectiveness of public health responses (9). Therefore, understanding and fostering the factors that can sustain this workforce is not merely an operational concern but a strategic imperative for maintaining health system functionality and broader societal stability during and after crises.

### 1.2 Theoretical framework and literature review: navigating turnover through organizational and psychological resources

The intention to leave one’s job, or turnover intention, is a significant predictor of actual employee turnover and has been extensively studied across various sectors, including healthcare (10). It represents a critical juncture where an employee cognitively and emotionally disengages, leading to detrimental consequences such as increased recruitment and training costs, loss of organizational knowledge, reduced quality of care, and diminished team morale (11). In the high-stakes environment of public health crises, mitigating turnover intention among community GPs is thus of utmost importance.

The Job Demands-Resources (JD-R) model provides a robust theoretical lens through which to understand the dynamics of employee well-being and motivation, particularly in demanding work environments (12). The model posits that job strain and burnout result from an imbalance where high job demands (e.g., workload, emotional pressure during a crisis) are not adequately buffered by job resources (13). Conversely, job resources—physical, psychological, social, or organizational aspects of the job that are functional in achieving work goals, reducing job demands, and stimulating personal growth and development—can foster work engagement and buffer the negative impact of job demands (14).

Among such outcomes, work engagement—a positive, fulfilling work-related state characterized by vigor, dedication, and absorption—is of particular relevance (15). A wealth of research has demonstrated that engaged employees exhibit higher performance, greater organizational commitment, and notably, lower turnover intentions (16). In the context of crisis-stricken healthcare settings, fostering work engagement can be a crucial psychological resource empowering GPs to navigate immense pressures and maintain their commitment (17). However, the antecedents of work engagement, especially organizational-level resources during crises, require further elucidation.

A key yet understudied construct in this context is organizational resilience. This refers to an organization’s capacity to anticipate, respond to, and adapt effectively in the face of disruptions while preserving core functions and values (18). In healthcare settings, resilient organizations typically feature strong leadership, adaptive workflows, transparent communication, supportive climates, and the ability to learn from adversity (19). These attributes can operate as organizational-level job resources that enhance employee engagement and retention (20). For example, a well-coordinated pandemic response with timely communication and leadership visibility can reduce uncertainty (a job demand), while fostering psychological safety and morale among staff.

In parallel, the concept of psychological contract adds a valuable layer to understanding the employee–organization relationship during crises. The psychological contract refers to an individual’s beliefs regarding mutual obligations between themselves and the organization, encompassing perceived expectations of support, fairness, recognition, and safety (20). When organizations demonstrate resilience—by ensuring resource adequacy, protecting staff, and maintaining open communication—they help uphold these implicit expectations. Conversely, breaches in the psychological contract, such as unfulfilled promises of support during crises, may erode engagement and increase turnover intentions. Thus, perceived organizational resilience may not only serve as a structural buffer, but also preserve the psychological contract, reinforcing motivation and loyalty during turbulent times.

While the theoretical importance of both organizational resilience and psychological contract is increasingly acknowledged, empirical studies directly examining how perceived resilience fosters work engagement and reduces turnover intention in frontline primary care settings remain scarce. Particularly lacking are models that integrate these constructs to explain how organizational features translate into psychological mechanisms and behavioral outcomes. This study addresses that gap by empirically testing a mediational pathway through which perceived organizational resilience influences turnover intention via work engagement, guided by the JD-R model and enriched by the psychological contract perspective.

### 1.3 The present study: aims and hypotheses

**Figure 1:**
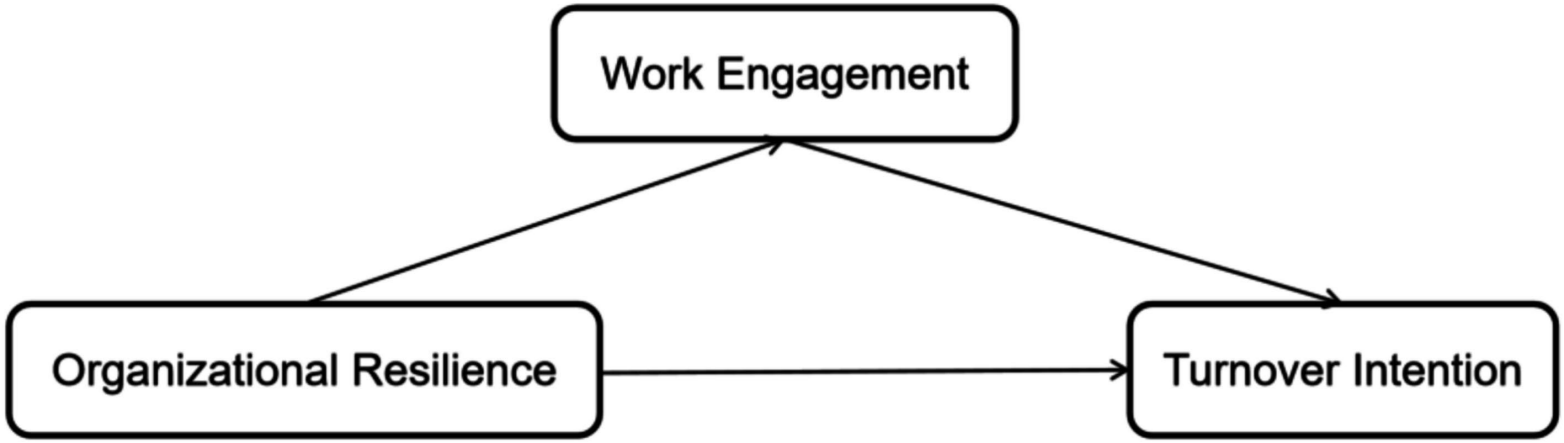
The theoretical model.

The present study was conducted among community GPs in Luohu District, Shenzhen, China, a region where all community health centers are governed by a unified hospital group, providing a unique context to examine organizational-level influences (21). Against the backdrop of experiences from public health crises (such as the COVID-19 pandemic), this research aims to investigate the interplay between organizational resilience, work engagement, and turnover intention among community GPs. Based on the JD-R model and the reviewed literature, the following hypotheses were formulated (Figure 1):

*H1:* Perceived organizational resilience will be negatively associated with turnover intention among community GPs.

*H2:* Perceived organizational resilience will be positively associated with work engagement among community GPs.

*H3:* Work engagement will be negatively associated with turnover intention among community GPs.

*H4:* Work engagement will mediate the relationship between perceived organizational resilience and turnover intention among community GPs.

### 1.4 Significance of the study

This study is poised to make several important contributions. Theoretically, it will extend the application of the JD-R model by examining organizational resilience as a critical organizational resource and work engagement as a key psychological mechanism influencing turnover intention in the unique and highly demanding context of community health services during crises. It aims to provide empirical evidence on the interplay of these constructs, particularly highlighting the mediating role of work engagement, which has been underexplored in this specific population and setting.

Practically, the findings are expected to offer valuable insights for healthcare administrators, policymakers, and hospital group managers. By identifying the protective role of organizational resilience and work engagement, this research can inform the development of targeted, cost-effective, and non-technological strategies to strengthen the primary healthcare workforce. Such strategies are crucial not only for retaining experienced GPs and ensuring the continuity and quality of community health services during future public health emergencies but also for fostering a more sustainable and responsive health system overall. Ultimately, a resilient and engaged primary healthcare workforce is fundamental to broader goals of societal well-being, economic stability during crises, and effective long-term pandemic preparedness. The insights gained from Chinese general practitioners can also offer transferable lessons for other health systems globally facing similar challenges in workforce retention and crisis management.

## 2 Materials and methods

### 2.1 Study design

This study adopted a cross-sectional survey design to investigate the relationships among perceived organizational resilience, work engagement, and turnover intention among community general practitioners (GPs). It further examined the mediating role of work engagement in this relationship. Data collection was conducted from August to December 2024, a period following the COVID-19 pandemic, during which China’s healthcare system underwent significant structural stress and recovery. Although the immediate crisis had subsided, many frontline healthcare workers continued to experience residual effects such as high workload, resource strain, and psychological fatigue—representing a prolonged post-crisis context relevant for evaluating organizational resilience and workforce stability. This context provides a meaningful backdrop for assessing how healthcare organizations support their employees in navigating lingering uncertainty and system-wide recovery.

### 2.2 Study setting and participants

#### 2.2.1 Study setting

The research was conducted in Luohu District of Shenzhen, a major urban center in southern China known for its rapid development and innovation in public health system reform. Luohu has played a pioneering role in establishing an integrated healthcare delivery system. All Community Health Centers (CHCs) in the district are governed by a single administrative entity—the Luohu Hospital Group—formed as part of China’s national initiative to enhance primary care and improve response capacity to public health emergencies. This centralized and unified governance model provides a rare, consistent organizational environment that minimizes variability in management structures and policy implementation. It thus serves as an ideal setting for examining the impact of perceived organizational-level factors, such as organizational resilience, on psychological and behavioral outcomes in primary healthcare workers. Moreover, during the COVID-19 pandemic and in its aftermath, Luohu’s CHCs served as the first line of response, responsible for mass testing, vaccination, public health communication, and the continuation of chronic disease care—further solidifying their role as frontline responders in crisis management. This setting therefore represents a high-relevance, high-intensity organizational environment in which to study the sustainability of primary care workforces under prolonged stress.

#### 2.2.2 Study participants and sampling

The target population for this study comprised general practitioners (GPs) employed in the Community Health Centers (CHCs) affiliated with the Luohu Hospital Group. Due to the centralized management system of the Luohu Hospital Group, which oversees all CHCs in the district under a unified administrative framework, a census sampling approach was employed to maximize representativeness and inclusivity.

According to administrative records, approximately 1,020 registered GPs were working across all CHCs at the time of the study. Based on this figure, 1,000 questionnaires were distributed to eligible participants, accounting for potential non-response or ineligibility. This approach ensured that the entire target population was approached, enhancing the generalizability of the findings. The final valid sample consisted of 927 completed questionnaires (after excluding those with more than 15% missing data or patterned responses), yielding a high response rate of 92.7%, which reflects both strong institutional coordination and participant engagement.

Inclusion Criteria: (1) Registered GPs actively practicing in CHCs under the Luohu Hospital Group; (2) Possession of a valid physician practice license; (3) At least 6 months of continuous tenure at their current CHC; (4) Voluntary participation with informed consent.

Exclusion Criteria: (1) Interns, resident physicians in standardized training, or temporary staff; (2) GPs absent from their post for more than 1 month during the data collection period due to leave or reassignment; (3) Incomplete or invalid responses on core variables.

### 2.3 Ethical considerations

The study complied with the Declaration of Helsinki. Approval was obtained from the Ethics Committee of Shenzhen Luohu People’s Hospital (Approval No.: 2024-LHQRMYY-KYLL-058). All participants were informed of the study’s purpose, procedures, risks, benefits, and their right to withdraw. Written informed consent was obtained, and anonymity and confidentiality were strictly maintained. Data were used solely for research purposes and reported in aggregate form.

### 2.4 Data collection and measures

#### 2.4.1 Data collection procedure

Data collection was conducted between August and December 2024. A multi-mode distribution strategy was implemented to accommodate varying work schedules and preferences of participants. Paper-based questionnaires were the primary mode, delivered and collected by trained research assistants or designated coordinators at each CHC. To reduce data loss due to logistical barriers (e.g., leave, offsite training), a secure online survey platform (using a password-protected link) was used to supplement data collection when necessary. Of the 927 valid questionnaires, 132 (14.2%) were completed via the secure online platform, while the remaining 795 (85.8%) were collected through paper-based formats. All participants completed the questionnaire independently. For paper surveys, responses were returned in sealed envelopes deposited into secure collection boxes to ensure anonymity. Data were reviewed for completeness and accuracy by the research team before analysis. To ensure data quality, double data entry and logical consistency checks were performed.

#### 2.4.2 Measures

Demographic and occupational information: included age, gender, marital status, education, income, employment status (permanent or contract), professional title, type of job, work years, and perceived public health workload, frequency of patient complaints.

Organizational resilience: organizational resilience was operationalized as GPs’ perceived capacity of their employing institution to effectively anticipate, respond to, and adapt to disruptions while maintaining essential functions. Measured using Zhang Xiu’e’s 15-item scale covering adaptability (6 items), foresight (5 items), and situational awareness (4 items), rated on a 5-point Likert scale (22). Higher scores indicated greater perceived resilience. The scale showed good reliability and validity. The Cronbach’s *α* in this study was 0.864.

Work engagement: work engagement was defined as a positive, fulfilling, work-related psychological state characterized by high levels of energy, dedication, and immersion in one’s work. It was measured using the Chinese version of the 16-item Utrecht Work Engagement Scale (UWES), comprising vigor (6 items), dedication (5 items), and absorption (5 items) (23). Responses used a 7-point Likert scale (0 = never, 6 = always). Higher scores indicated stronger work engagement. The Cronbach’s *α* in this study was 0.864, subscales ranged from 0.902 to 0.953.

Turnover intention: turnover intention was conceptualized as an individual’s conscious and deliberate desire to leave their current job, serving as a strong proximal predictor of actual resignation. Measured using the 6-item scale revised by Li Dongrong and Li Jingyuan (24), with three subscales: possibility of turnover, motivation to seek another job, and perceived likelihood of obtaining another job. Rated on a 4-point Likert scale (1 = strongly disagree, 4 = strongly agree). Higher scores indicated stronger turnover intention. The Cronbach’s *α* in this study was 0.799.

Control variables: based on findings from previous relevant literature and preliminary univariate analyses of the current study’s data, the following variables were included as covariates in the statistical models to more accurately assess the relationships between the core variables: Age,gender, monthly income level, employment status, professional title, and perceived public health workload were included as covariates (25, 26).

### 2.5 Statistical analysis

Data were double-entered using EpiData 3.1 and analyzed in SPSS 26.0 (IBM Corp., Armonk, NY, USA). Means, standard deviations, frequencies, and percentages were calculated for all variables. Harman’s single-factor test was conducted to detect potential common method bias. Pearson correlation coefficients were computed to explore associations among organizational resilience, work engagement, and turnover intention. Mediation effects were tested using the Bootstrap method via Hayes’ PROCESS V3.5 macro (Model 4), with 5,000 resamples to generate a 95% bias-corrected confidence interval. Mediating effect was considered significant if the confidence interval did not contain zero. Two-tailed *p*-value less than 0.05 was considered statistically significant.

## 3 Results

### 3.1 Descriptive statistics and common method bias test

A total of 927 valid questionnaires were analyzed, yielding an effective response rate of 92.7%. Participants were nearly evenly split by gender (49.41% male, 50.59% female), with the majority aged 30–40 years (33.23%) or younger than 30 (33.76%). Over half of the GPs held junior or intermediate professional titles, and nearly half (49.51%) were contract employment status physicians. To assess potential common method bias due to the use of self-report questionnaires, Harman’s single-factor test was performed. The first factor accounted for 28.297% of the variance, which was below the critical threshold of 40%, suggesting no significant common method bias.

### 3.2 Differences in turnover intention across demographic variables

Table 1 presented the results of univariate analyses. Turnover intention differed significantly by age (*F* = 29.55, *p* < 0.001), professional title (*F* = 28.22, *p* < 0.001), cadre status (*t* = −6.81, *p* < 0.001), monthly income (*F* = 29.89, *p* < 0.001), and perceived basic public health workload (*F* = 34.40, *p* < 0.001). GPs who were ≤30 years old, without professional title, non-cadre, earning ≤8,000 yuan/month, or reporting heavy public health workload exhibited significantly higher turnover intentions.

**Table 1:** Demographic differences in GPs turnover intention.

Variables	*n* (%)	Turnover, Mean ± SD	*t/F*	*p*
Gender			t = 0.84	0.40
Male	458 (49.41)	15.16 ± 5.51		
Female	469 (50.59)	14.85 ± 5.65		
Age (Years)			*F* = 29.55	**<0.001**
≤30	313 (33.76)	16.84 ± 5.28		
31 ~ 40	308 (33.23)	14.55 ± 5.49		
≥41	306 (33.01)	13.58 ± 5.47		
Marital Status			*F* = 1.37	0.26
Unmarried	318 (34.30)	15.31 ± 5.72		
Married	324 (34.95)	14.60 ± 5.45		
Others	285 (30.74)	15.11 ± 5.54		
Education			*F* = 1.64	0.19
College degree or below	325 (35.06)	15.38 ± 5.38		
Undergraduate	308 (33.23)	14.57 ± 5.61		
Master degree or above	294 (31.72)	15.04 ± 5.74		
Profession title			*F* = 28.22	**<0.001**
No title	245 (26.43)	16.52 ± 5.30		
Primary	218 (23.52)	15.97 ± 5.37		
Intermediate	235 (25.35)	15.14 ± 5.19		
Senior	229 (24.70)	12.32 ± 5.54		
Work Years			*F* = 0.21	0.89
≤5	244 (26.32)	15.06 ± 5.77		
6 ~ 10	228 (24.60)	15.21 ± 5.42		
11 ~ 20	238 (25.67)	14.92 ± 5.76		
≥21	217 (23.41)	14.82 ± 5.34		
Employment status			*t* = −6.81	**<0.001**
Permanent	468 (50.49)	13.80 ± 5.34		
Contract	459 (49.51)	16.23 ± 5.55		
Monthly Income(Yuan)			*F* = 29.89	**<0.001**
≤8,000	212 (22.87)	17.11 ± 5.24		
8,001 ~ 12,000	237 (25.57)	16.16 ± 5.16		
12,001 ~ 15,000	230 (24.81)	14.17 ± 5.41		
>15,000	248 (26.75)	12.87 ± 5.51		
Basic public health services burden			*F* = 34.40	**<0.001**
Minimal	314 (33.87)	13.26 ± 5.39		
Occasional	325 (35.06)	14.99 ± 5.39		
Heavy	288 (31.07)	16.91 ± 5.37		
Frequency of patient complaints			*F* = 0.67	0.57
Seldom	213 (22.98)	15.36 ± 5.56		
Occasional	232 (25.03)	14.70 ± 5.60		
Often	248 (26.75)	15.15 ± 5.42		
Very frequent	234 (25.24)	14.82 ± 5.75		
Type of job			*F* = 0.26	0.94
General out patient service	153 (16.50)	15.05 ± 5.71		
Family doctor contract service	150 (16.18)	15.02 ± 5.76		
Chronic disease management	167 (18.02)	15.27 ± 5.01		
Management of public health	147 (15.86)	15.18 ± 5.58		
Administration	150 (16.18)	14.82 ± 6.05		
Others	160 (17.26)	14.67 ± 5.43		

“Title” refers to professional ranks of Chinese physicians. “No Title” = physicians without formal rank; “Primary” = junior-level titles such as Resident Physician; “Intermediate” = mid-level titles such as Attending Physician; “Senior” = senior-level titles such as Chief or Associate Chief Physician. SD, standard deviation t, t-test, F, ANOVA. Bold indicates that the statistical test difference of the variable is significant.

### 3.3 Correlations among key variables

Descriptive statistics and Pearson correlation coefficients were presented in Table 2. Organizational resilience (*M* = 45.21, SD = 14.89), work engagement (*M* = 48.32, SD = 22.96), and turnover intention (*M* = 15.02, SD = 5.57) were significantly correlated. Specifically, organizational resilience was negatively associated with turnover intention (*r* = −0.288, *p* < 0.001) and positively associated with work engagement (*r* = 0.238, *p* < 0.001). Work engagement was also negatively correlated with turnover intention (*r* = −0.264, *p* < 0.001).

**Table 2:** Description statistics and correlation analysis of each variable.

Variables	M ± SD	1	2	3
1. Organizational Resilience	45.21 ± 14.89	1		
2. Work Engagement	48.32 ± 22.96	0.238**	1	
3. Turnover Intention	15.02 ± 5.57	−0.288**	−0.264**	1

^**^p<0.01. SD: Standard Deviation.

### 3.4 Mediation analysis: work engagement as a mediator

Hierarchical regression and mediation analysis were conducted to test the hypothesized relationships (see Tables 3, 4). In Step 1, organizational resilience significantly predicted lower turnover intention (*β* = −0.287, *p* < 0.001). In Step 2, organizational resilience significantly predicted higher work engagement (*β* = 0.238, *p* < 0.001). In Step 3, both organizational resilience (*β* = −0.238, *p* < 0.001) and work engagement (*β* = −0.207, *p* < 0.001) were significant predictors of turnover intention, suggesting partial mediation. Bootstrapping results indicated a significant indirect effect of organizational resilience on turnover intention via work engagement (*B* = −0.018, SE = 0.003, 95% CI [−0.070, −0.011]), accounting for 16.82% of the total effect.

**Table 3:** Summary of hierarchical regression analyses predicting turnover intention.

Regression equation		Overall fit coefficient			Regression coefficient			
Outcome variables	Predictor variables	*R*	*R^2^*	*F*	*β*	*B*	*SE*	*t*
Turnover intention	Organizational resilience	0.287	0.082	89.442**	−0.287	−0.107	0.011	−9.134**
Work engagement	Organizational resilience	0.238	0.057	55.647**	0.238	0.367	0.049	7.459**
Turnover intention	Organizational resilience	0.351	0.123	64.920**	−0.238	−0.089	0.012	−7.513**
	Work engagement				−0.207	−0.050	0.007	−6.530**

**p<0.01. CI, Confidence Interval, SE, Standard Error, β, Standardized Regression Coefficient, B, Unstandardized Regression Coefficient, R^2^, Coefficient of Determination.

**Table 4:** Direct and indirect effects of organizational resilience on turnover intention.

Path	*B*	*SE*	95%CI	Ratio of effect values
Total effect	−0.107	0.011	[−0.131, −0.084]	
Direct effect	−0.089	0.012	[−0.112, −0.659]	83.18%
Indirect effect	−0.018	0.003	[−0.070, −0.011]	16.82%

### 3.5 Sensitivity analysis with covariates

To assess the robustness of the mediation effect, sensitivity analysis was conducted controlling for age, professional title, employment status, monthly income, and perceived public health service burden (see Tables 5, 6). After controlling for covariates, organizational resilience remained a significant negative predictor of turnover intention (*β* = −0.246, *p* < 0.001), and work engagement remained a significant mediator (*β* = −0.214, *p* < 0.001). The adjusted indirect effect was significant (*B* = −0.019, SE = 0.003, 95% CI [−0.026, −0.012]), and accounted for 17.12% of the total effect.

**Table 5:** Summary of hierarchical regression analyses predicting turnover intention.

Regression equation		Overall fit coefficient			Regression coefficient			
Outcome variables	Predictor variables	*R*	*R^2^*	*F*	*β*	*B*	*SE*	*t*
Turnover intention	Organizational resilience	0.648	0.420	111.215**	−0.297	−0.111	0.009	25.330**
	Age				−0.234	−1.601	0.171	−11.836**
	Title				−0.277	−1.373	0.124	−9.339**
	Employment status				0.211	2.356	0.281	8.367**
	Monthly income				−0.287	−1.438	0.126	−11.361**
	Basic public health services burden				0.277	1.923	0.174	11.023**
Work engagement	Organizational resilience	0.244	0.059	9.709**	0.237	0.365	0.049	7.414**
	Age				−0.035	−0.984	0.899	−1.094
	Title				0.024	0.501	0.653	0.767
	Employment status				−0.023	−1.095	1.476	−0.742
	Monthly income				−0.016	−0.334	0.663	−0.504
	Basic public health services burden				0.016	0.477	0.914	0.521
Turnover intention	Organizational resilience	0.680	0.463	113.422**	−0.246	−0.092	0.009	−9.904**
	Work engagement				−0.214	−0.052	0.006	−8.592**
	Age				−0.242	−1,652	0.165	−10.005**
	Title				−0.272	−1.347	0.120	−11.228**
	Employment status				0.206	2.299	0.271	8.479**
	Monthly income				−0.290	−1.456	0.121	−11.948**
	Basic public health services burden				0.281	1.948	0.168	11.597**

**p<0.01. CI, Confidence Interval, SE, Standard Error, β, Standardized Regression Coefficient, B, Unstandardized Regression Coefficient, R^2^, Coefficient of Determination.

**Table 6:** Direct and indirect effects of organizational resilience on turnover intention.

Path	*B*	*SE*	95%CI	Ratio of effect values
Total effect	−0.111	0.009	[−0.129, −0.092]	
Direct effect	-0.092	0.009	[−0.110, −0.074]	82.88%
Indirect effect	−0.019	0.003	[−0.026, −0.012]	17.12%

These findings suggested that the mediating effect of work engagement between organizational resilience and turnover intention was robust and stable even after adjusting for key demographic and occupational variables.

## 4 Discussion

### 4.1 Summary of main findings

This study investigated turnover intention among community general practitioners in Luohu District, Shenzhen. Results showed high prevalence of turnover intention. Organizational resilience was negatively associated with turnover intention and positively linked to work engagement. Work engagement also negatively predicted turnover intention and partially mediated the resilience-turnover relationship. These associations remained significant after adjusting for demographic and occupational variables, highlighting the importance of organizational and psychological factors in workforce retention. Taken together, the findings provide empirical support for all four hypothesized relationships proposed in this study.

### 4.2 Turnover intention status and its influencing factors among community GPs

#### 4.2.1 Overall level and severity of turnover intention

The mean turnover intention score among community GPs was 15.02. Based on scale criteria, 36.25% reported “very high” and 28.91% reported “high” turnover intention, totaling 65.16% with elevated departure risk. This high prevalence signals a serious threat to workforce stability in community healthcare (27). Sustained high turnover may disrupt care continuity, reduce service quality, and impair primary healthcare system efficiency (28, 29). These findings highlight the urgent need for targeted interventions to mitigate turnover drivers and strengthen retention in frontline health services.

#### 4.2.2 Demographic and occupational differences in turnover intention

Univariate analyses (Table 1) revealed significant demographic and occupational disparities in turnover intention. Younger GPs (≤30 years), those without professional titles, non-cadre employees, low-income earners (≤8,000 yuan/month), and those reporting heavy public health workloads all exhibited significantly higher intention to leave. These trends are consistent with prior research showing early-career physicians are more mobile (30), financially constrained staff face greater job dissatisfaction (31), and excessive workload contributes to burnout (32).

Particularly, cadre status and professional rank reflected systemic disparities in employment stability and advancement opportunities. GPs with permanent posts and senior titles showed markedly lower turnover intention, underscoring the role of job security and career recognition (33). Collectively, these findings highlight high-risk subgroups within the community GP workforce. Retention strategies must be multifaceted—addressing not only organizational culture but also employment terms, compensation equity, workload distribution, and early-career support. Tailored interventions for these vulnerable groups are essential for sustaining a stable and motivated primary healthcare workforce.

### 4.3 Discussion of core hypotheses

This study empirically supported all four core hypotheses, illuminating the interplay between organizational resilience, work engagement, and turnover intention among community GPs.

#### 4.3.1 Organizational resilience and turnover intention

The confirmation of H1—that higher perceived organizational resilience predicts lower turnover intention—aligns with existing literature emphasizing the retention benefits of stable, supportive workplaces (34). When GPs perceive their organization as adaptive and prepared for adversity, they are more likely to experience psychological safety and reduced uncertainty. This mitigates stress that often triggers turnover thoughts, particularly in high-demand healthcare environments (35). Within the Job Demands–Resources (JD-R) model (36), organizational resilience serves as a crucial resource that buffers job stressors, thereby reducing turnover motivation. Thus, the observed inverse association between organizational resilience and turnover intention confirms Hypothesis 1.

#### 4.3.2 Organizational resilience and work engagement

H2, which posited a positive relationship between organizational resilience and work engagement, was also supported. Resilient organizations likely cultivate climates of trust, adaptability, and psychological safety—key conditions for sustained employee engagement (37). GPs in such environments are more likely to experience vigor, dedication, and absorption in their work. According to the JD-R model, organizational resilience activates the motivational pathway by providing necessary resources, reinforcing the idea that system-level strength contributes directly to individual engagement. The significant positive correlation between organizational resilience and work engagement confirms Hypothesis 2.

#### 4.3.3 Work engagement and turnover intention

H3, confirming the negative association between work engagement and turnover intention, aligns with extensive evidence across occupational sectors (38). GPs who feel energized and purpose-driven are more affectively bonded to their roles and organizations. These positive experiences reduce the appeal of alternative employment and reinforce retention. Our findings thus validate Hypothesis 3, demonstrating that higher work engagement is associated with lower turnover intention. This highlights the strategic value of promoting work engagement as a protective factor against workforce attrition.

#### 4.3.4 The mediating role of work engagement

H4 was supported, confirming that work engagement partially mediated the relationship between perceived organizational resilience and turnover intention. This result provides important insight into the psychological mechanism by which organizational-level characteristics influence individual behavior. It is not merely the presence of resilience that matters—but the way it enhances the employee’s experience of work, specifically their engagement, that influences retention decisions. The partial mediation confirmed in our analysis supports Hypothesis 4 and illustrates how organizational resilience affects turnover intention through the motivational pathway of engagement.

Organizational resilience, marked by attributes such as effective crisis management, adaptive leadership, and proactive communication, likely fosters an environment of psychological safety, predictability, and trust (39). These conditions enable GPs to invest more energy into their work (vigor), feel greater purpose (dedication), and become more immersed in their tasks (absorption), rather than being distracted by uncertainty. Such positive engagement experiences serve as strong intrinsic motivators that reduce the attractiveness of leaving.

From the JD-R perspective, organizational resilience operates as a bundled job resource that fuels the motivational process underlying work engagement (40). Engaged employees are more likely to maintain positive attitudes and exhibit lower turnover intentions. The mediation observed was partial, suggesting additional pathways—such as enhanced job security or reduced role ambiguity—may also directly link resilience to retention. Future studies could further explore these complementary mechanisms. In summary, the study confirms all four hypotheses, with results supporting a pathway from organizational resilience to reduced turnover intention, both directly and indirectly via enhanced work engagement.

Practically, this finding suggests that interventions to reduce GP turnover must not only enhance structural resilience but also ensure that such resilience translates into meaningful improvements in daily work experience. Resilient systems alone are insufficient if they fail to foster employee engagement. Therefore, health leaders should combine structural improvements with active engagement strategies—such as promoting autonomy, recognition, and professional growth—embedded within a resilient organizational culture.

### 4.4 Theoretical implications

This study contributes to the literature in three key ways. First, it extends the Job Demands–Resources (JD-R) model by empirically validating organizational resilience as a critical organizational resource within the high-demand context of Chinese community general practice. The demonstrated pathway—resilience enhancing engagement, which in turn reduces turnover intention—adds clarity to the interplay between organizational and individual-level factors in shaping employee behavior. Second, it adds to emerging healthcare research on organizational resilience. While previous studies often emphasized individual resilience, our findings underscore the independent value of organizational-level resilience in supporting workforce stability. An organization’s ability to anticipate, respond to, and adapt to adversity significantly impacts employee engagement and retention. Third, by identifying work engagement as a key mediator, this study illuminates a specific psychological mechanism through which resilience operates. Rather than assuming a direct relationship, it shows how macro-level capacities influence micro-level experiences, offering a more nuanced understanding of employee outcomes in high-stress settings. Together, these findings strengthen theoretical models of turnover and support a multi-level framework for understanding how resilient systems promote sustainable healthcare workforce outcomes.

### 4.5 Practical implications and policy recommendations

This study offers several actionable insights for improving the retention of community general practitioners. To begin with, healthcare organizations should prioritize the development of organizational resilience. Key strategies include strengthening crisis response systems, promoting adaptive leadership, improving resource availability, and cultivating a learning-oriented, communicative culture. These efforts should address the core dimensions of resilience: adaptability, foresight, and situational awareness. Equally important is the need to foster work engagement. Enhancing job autonomy and variety, offering meaningful feedback and recognition, and supporting professional development can help cultivate a more motivated and committed workforce. Notably, such engagement strategies are most effective when implemented within a resilient organizational environment. In addition, disparities identified across demographic groups point to the necessity of targeted support. Early-career GPs, those without professional titles, non-cadre staff, and those under heavy public health workload would benefit from tailored interventions such as mentorship programs, clearer promotion pathways, compensation reviews, and stress management initiatives. Integrating resilience-building with engagement promotion may yield compounded benefits. For instance, clear communication during crises not only stabilizes organizational functioning but also reduces uncertainty and enhances staff engagement. These findings underscore that effective GP retention depends on simultaneously strengthening organizational systems and addressing the psychological needs of frontline professionals.

### 4.6 Strengths and limitations of the study

This study has several strengths. A large sample size ensured sufficient statistical power, and focusing on GPs within a single, uniformly managed hospital group (Luohu Hospital Group) minimized inter-organizational confounding. The use of validated scales and rigorous analyses—including bootstrapped mediation testing and covariate adjustment—enhanced the robustness of findings.

However, several limitations must be acknowledged. First, the study employed a cross-sectional design, which restricts causal inference despite the theory-driven model and temporal logic embedded in the hypotheses. Second, all measures were based on self-report, which may introduce common method variance and social desirability bias; although Harman’s single-factor test suggested minimal common-method bias, these risks cannot be fully excluded. Third, the study was conducted in a single, economically developed urban district in China, which may limit the generalizability of the findings to other regions or health systems, especially in rural or decentralized contexts. Fourth, while the study measured turnover intention, which is a well-established predictor of actual turnover behavior, it is not equivalent to measuring actual turnover, and some of the literature used for comparison may have relied on observed attrition data. Lastly, other unmeasured factors—such as personality traits, specific leadership styles, or past exposure to crisis events—may have influenced the observed associations and should be examined in future research.

### 4.7 Directions for future research

Future studies should employ longitudinal designs to confirm causal pathways and examine how organizational resilience and engagement evolve across time and stress phases. Intervention trials could evaluate strategies to enhance resilience and engagement in community health settings. Further exploration of additional mediators and moderators—such as psychological capital, organizational trust, or leadership behavior—is warranted. Comparative research across diverse regions, professions, and health systems would help test generalizability. Qualitative approaches, including interviews with GPs, could also offer deeper insight into how they perceive organizational resilience and make career decisions.

## 5 Conclusion

This study highlights the critical issue of high turnover intention among community general practitioners in Shenzhen, with nearly two-thirds expressing a desire to leave. Organizational resilience emerged as a key protective factor, indirectly reducing turnover intention by enhancing work engagement. These effects remained robust after adjusting for key demographic and occupational variables. Strengthening organizational resilience and fostering engagement represent actionable strategies for healthcare leaders seeking to stabilize the primary care workforce and ensure the continuity and quality of community health services in challenging environments.

## Data Availability

The raw data supporting the conclusions of this article will be made available by the authors, without undue reservation.
